# Supplementary figures and images for: YB-1 Is Altered in Pregnancy-Associated Disorders and Affects Trophoblast in Vitro Properties via Alternation of Multiple Molecular Traits
Source: Int J Mol Sci. 2021 Jul 5;22(13):7226. doi: 10.3390/ijms22137226 (PMC8269420; doi:10.3390/ijms22137226)

A

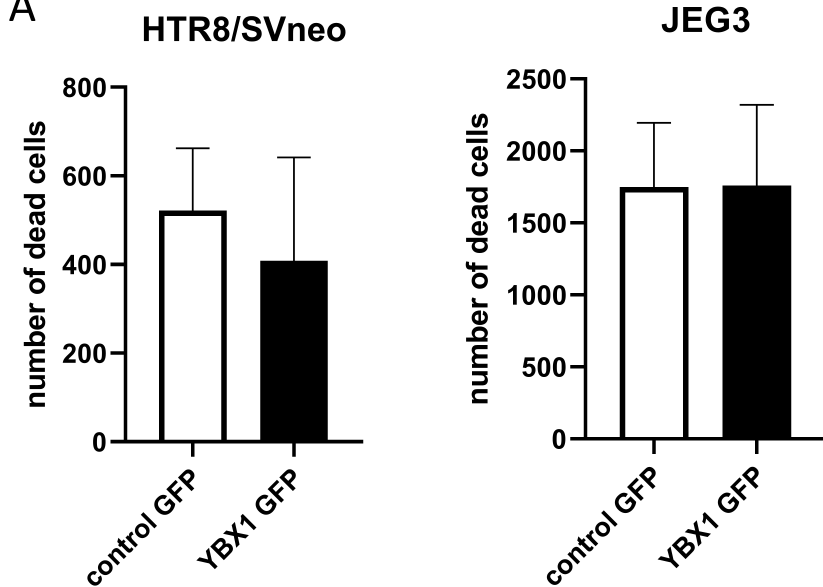

B

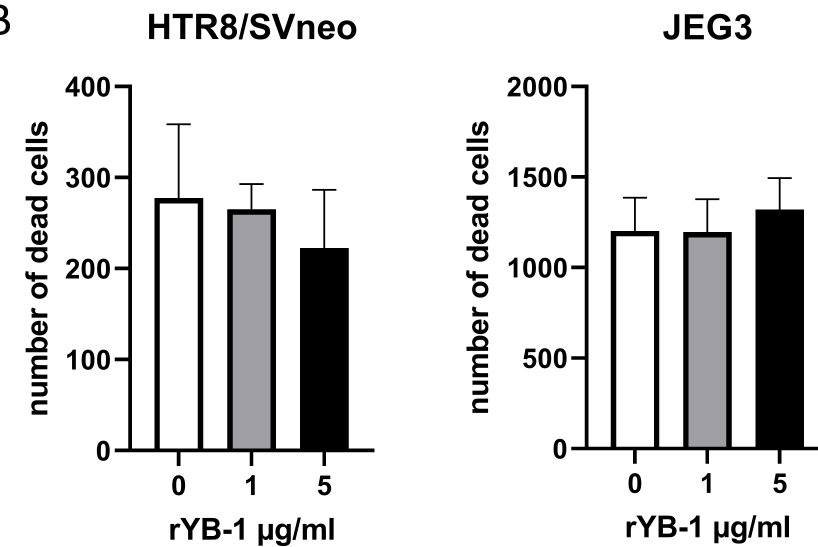

Supplement: Supplementary file 1 [file ijms-22-07226-s001.zip › ijms-1268851-suppl.pdf]
